# Influence of Self-Relevance and Reputational Concerns on Altruistic Moral Decision Making

**DOI:** 10.3389/fpsyg.2019.02194

**Published:** 2019-09-26

**Authors:** Youlong Zhan, Xiao Xiao, Qianbao Tan, Shangming Zhang, Yangyi Ou, Haibo Zhou, Jin Li, Yiping Zhong

**Affiliations:** ^1^Department of Psychology, Hunan University of Science and Technology, Xiangtan, China; ^2^College of Chengnan, Hunan First Normal University, Changsha, China; ^3^Department of Psychology, Hunan Normal University, Changsha, China

**Keywords:** moral decision making, self-relevance, reputational concerns, egoistic, altruistic

## Abstract

Complex moral decision making may share certain cognitive mechanisms with economic decision making under risk situations. However, it is little known how people weigh gains and losses between self and others during moral decision making under risk situations. The current study adopted the dilemma scenario-priming paradigm to examine how self-relevance and reputational concerns influenced moral decision making. Participants were asked to decide whether they were willing to sacrifice their own interests to help the protagonist (friend, acquaintance, or stranger) under the dilemmas of reputational loss risk, while the helping choices, decision times and emotional responses were recorded. In Study 1, participants showed a differential altruistic tendency, indicating that participants took less time to make more helping choices and subsequently reported weaker unpleasant experience toward friends compared to acquaintances and strangers. In Study 2, participants still made these egoistically biased altruistic choices under the low reputational loss risk conditions. However, such an effect was weakened by the high reputational loss risks. Results suggested that moral principle guiding interpersonal moral decision making observed in our study is best described as an egoistically biased altruism, and that reputational concerns can play a key role in restraining selfish tendency.

## Introduction

How we make decisions that have direct consequences for ourselves and others forms the moral foundation of our society (Volz et al., 2017). Economists typically characterized humans as profoundly selfish in economic decisions, but some recent psychological studies showed that people were hyperaltruistic in some social decisions, such as moral decision making (Crockett et al., 2014, 2015; Volz et al., 2017). Moral decision making is supposed as the ability to choose an optimal course of action among multiple alternatives within a system of norms and values that guides our behaviors in a community (Rilling and Sanfey, 2011). Generally, moral decision making usually involves a trade-off between maintaining personal benefits and preventing harm to others (Greene and Haidt, 2002; Pletti et al., 2015, 2016; Crockett, 2016; Szekeres et al., 2019). The tension between altruistic and egoistic tendencies strongly affected the behavioral responses and neural potentials during moral decision making. However, knowledge on how people make choices for gains and losses between self and others under different moral dilemmas is limited (Christensen and Gomila, 2012; Hughes, 2017).

In order to bridge this gap about how people weigh gains and losses between self and others during moral decision making, some researchers have sought to focus the critical role of self-relevance in this tension, which refers to whether moral stimuli or dilemmas are related to us personally or not (Christensen and Gomila, 2012; Sarlo et al., 2012). For example, people are more willing to sacrifice their self-interest to help family members and friends during moral judgments and decisions compared to strangers (Miller and Bersoff, 1998; Loke et al., 2011). The behavioral results of an ERP study have reported that participants made more helping choices toward their relatives compared to strangers (Chen et al., 2009). Additionally, recent seminal psychological studies suggested that people were instead hyperaltruistic, namely that people were more willing to forego more gains to reduce strangers’ harm than to spare themselves from harm (Crockett et al., 2014, 2015). However, participants showed strong egoistic tendencies in their unwillingness to harm themselves for strangers’ benefit (Volz et al., 2017). Therefore, these studies suggested that self-relevance indeed modulated the moral evaluation and judgment in the moral processing. Participants showed more egoistic tendencies toward the intimate persons during moral decision making compared with the distant persons (Christensen and Gomila, 2012).

Recently, human brain imaging studies found similar cognitive mechanisms and neural activations between moral and economic decision making in risky monetary situations (Palmer et al., 2013). For example, similar with studies on economic decision making, the venntro-media prefrontal cortex and ventral striatum were specifically sensitive to the expected moral value of options, and the right anterior insula was specifically sensitive to outcome probability during moral decision making (Tobler et al., 2008; Shenhav and Greene, 2010; Lee et al., 2018). Prescott (2012) indicated that participants were more inclined to avoid risks to make altruistic moral decision making under the positive frame contexts, while they were more inclined to seek risks to make egoistic moral decision making in the negative frame context. Moreover, they believed that prospect theory applied in economic decision making could also be used to explain the cognitive mechanism of human moral decision making. Additionally, Bixter and Luhmann (2014) have directly examined the influence of reputational loss risk on moral decision making. In detail, participants were found to make more risky decisions to gain more benefits for themselves under the anonymous conditions (lower risk), and such risky decisions for their benefits were reduced when the results of each decision were presented to the partner (higher risk). Recently, Arfer et al. (2015) have further revealed that participants who were told that their partners would see their choices were more prosocial during moral decision making, and suggested that reputational concerns were a key restraint on selfish exploitation under moral risk conditions. Therefore, information about reputational loss risk can modulate individual decision patterns and results during moral decision making.

Previous studies also explored the influence of self-relevance on risky decision making. Empirical studies indicated that deciding for others differed from deciding for the self (Polman, 2012). For example, Beisswanger et al. (2003) showed that individuals were riskier when decisions were made on behalf of others than oneself, whereas such difference was decreased when others had close relationships with decision makers. Garcia-Retamero and Galesic (2012) have reported that doctors made more risky predictions for unfamiliar patients compared to their familiar, whereas such difference of risk preference was not observed when doctors made the actual medical decisions for patients. Additionally, some studies reported that psychological closeness led individuals to make more risk-averse decisions, and psychologically remoteness led individuals to make more risk-seeking decisions (Zhao et al., 2013; Raue et al., 2015). Recently, Zhang et al. (2018) proposed the self-promotion hypothesis to explain the influence of self-relevance on risk decision making. This hypothesis suggested that risk decision making is not only a pure calculation activity around the probability of profit and loss, but also involves the projection and involvement of deeper desire and motivation. Therefore, risk decision making often needs to be weighed between obtaining gains and maintaining self-image. These findings tell us that people could make more risk-seeking decisions during economic decision making. However, it remains unclear regarding how self-relevance and external decision risk interact to impact individual moral decision making.

In conclusion, it matters who the other person is during moral decision making. Previous studies have operated self-relevance as the psychological distance which refers to subjective perception and emotional experience regarding interpersonal intimacy for different others (Agnew et al., 2004), such as specific and general others (Hsee and Weber, 1997), familiar and unfamiliar others (Stone and Allgaier, 2008 and Psychology), or similar and dissimilar others (Liviatan et al., 2008). However, these classifications of self-relevance are not enough. In the self-concept of Chinese people, interpersonal relationship presents a kind of differential pattern, reflecting that close others are in the core group around the self and alienated others are in the fringe group around the self (Zhu and Han, 2008; Ma and Han, 2011). Thus, the different intimate degree of self-relevance was usually operated as relatives (mother or father), friend, acquaintance, and stranger (Northoff et al., 2009; Fan et al., 2013; Tan et al., 2015; Zhan et al., 2016). Importantly, it is still necessary to explore how individuals make moral decisions in situations of interaction between self-relevance and external decision risk. Therefore, the present study first investigates the influence of different intimate degrees of self-relevance on moral decision making (Study 1) and further uncovers the modulation of reputational loss risk on this influence (Study 2). According to the influence of self-relevance on risky decision making, we hypothesize that individuals may make more altruistic choices for the high self-relevant others (i.e., friends) and take less decision time compared to the low self-relevant others (i.e., acquaintances or strangers) under the low reputational loss risk conditions. However, such differences may be decreased or disappear under high reputational loss risk conditions.

## Study 1

Study 1 was a single-factor within-subject experiment. In the moral dilemmas, we made self-relevance salient by telling participants that the protagonist of each dilemma was a friend, acquaintance, or stranger. We expected participants to make more altruistic choices in the high degree of self-relevant dilemmas (i.e., friend) compared to the low degree of self-relevant dilemmas (i.e., acquaintance and stranger).

### Methods

#### Participants

A power analysis (G^∗^Power 3.1; Faul et al., 2007) suggested that 36 participants would ensure 90% statistical power even in case of small-to-medium effect size (cf. Vazire, 2016). We recruited 72 participants (37 males, 35 females, average age 23.65 ± 0.86 years). All participants were right-handed and had normal or corrected-to-normal vision. Each participant gave written informed consent prior to participation and received 10 Yuan RMB ($1.45) for their reward after the experiment. The experiment was conducted in accordance with the Declaration of Helsinki and was also approved by the Ethics Committee of Hunan Normal University.

### Materials and Procedure

#### Moral Dilemmas

There were 30 common moral dilemmas involving costly help in daily life, which were quoted and adapted from previous studies about moral decision (Greene, 2009; Sarlo et al., 2012; Azevedo et al., 2017; Zhan et al., 2018). Each dilemma described the scenario in which a protagonist was in danger and needed help while the subject was going to do an important thing (e.g., he has a sudden illness and the subject is going to attend to a significant meeting). Meanwhile, two options (A and B) were represented to participants for choice. Option A described a hypothetical action of helping him/her at a cost and option B described an alternative hypothetical action of not helping him/her. Participants were instructed to carefully weigh the two options and to choose one. The numbers and familiarities of moral dilemmas were controlled and balanced between participants.

#### Procedure

**FIGURE 1:**
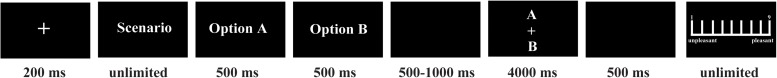
Sequence of events in the experiments of Study 1.

Firstly, upon arrival each participant was required to offer the names of a friend, acquaintance, and stranger as the protagonist in the dilemma. IOS was used to test whether the different degree of interpersonal intimacy was operated availably (Aron et al., 1992; Myers and Hodges, 2012). And then they were seated in a separated room and instructed on the experimental tasks. Secondly, participants were asked to complete the dilemma moral decision-making task (Sarlo et al., 2012, 2014; Wang et al., 2014; Pletti et al., 2015) while their behavioral responses (i.e., proportions of choices and response times) were recorded. The sequence of the task is displayed in Figure 1. In detail, a fixation cross was presented for 200 ms, and then the scenario of moral dilemma was presented with unlimited time until the participants pressed the keyboard to move to the next interface. Self-relevance was operated in the scenario by describing a protagonist (e.g., participant’s friend, acquaintance, or stranger) in danger and in need of timely help. And then Option A and B were presented, respectively, for 500 ms in a random order across trials. After a random blank for 500–1000 ms, a fixation cross appeared in the medium-term of the blank screen between the letters A and B for 4000 ms; the orientations were random across trials. Participants were asked to decide between the two options by pressing one of the two keys marked by letters A or B quickly and accurately. After a black blank was presented for 500 ms, participants were instructed to rate how they felt while they were deciding using a 9-point scale of affective valence (e.g., “how much displeasure do you feel when you were making the decision between two options?,” 1 = extremely unpleasant, 9 = extremely pleasant). Additionally, the visual angle of the decision slide was about 3.4° × 1.3° (the label A and B subtended 1.3° × 1.3°, the central fixation cross subtended 0.8° × 0.8° of visual angle). Participants were explicitly told to wait for the decision interface before evaluating the two options. All stimuli presentations were accomplished with E-prime software (Psychology Software Tools, Pittsburgh, PA, United States).

### Data Statistics and Analysis

According to the dual-process theory (Greene, 2009), moral decision making is usually involved with trade-offs between maintaining personal benefits and preventing harm to others, which evoked strong aversive emotion and moral conflict. Thus, we were interested in participants’ emotional responses (subjective unpleasure rating scores), cognitive process (decision times), and behavioral outcomes (helping proportions) during moral decision-making. Thus, we have recorded the proportions of helping choices, decision times, and the scores of IOS and subjective unpleasure rating scores in study 1. The participants with decision times exceeding plus or minus two standard deviations were excluded, and there was no participant to be excluded. ANOVA was used to analyze how these dependent variables were affected by the independent variables, namely self-relevance. Bonferroni-corrected test was used for the multiple post-average comparisons. SPSS 20.0 software was used to complete the statistics and analysis of all data.

### Results and Discussion

#### IOS Scores

The descriptive results (Average and standard deviations) of IOS scores, proportions of help choices, decision times and the subjective unpleasure rating scores are presented in Table 1. Firstly, we analyzed the results of the scores of IOS for target persons presented by each participant. ANOVA indicated that there was a significant main effect of self-relevance, *F*(2, 142) = 358.69, *p* < 0.001, $\eta_{p}^{2}$ = 0.89, suggesting that the IOS score between self and friend (*M* = 5.25, *SD* = 0.48) was higher than that between self and acquaintance (*M* = 3.35, *SD* = 0.50, *p* < 0.001, *d* = 3.88) and that between self and acquaintance was higher than that between self and stranger (*M* = 1.38, *SD* = 0.46, *p* < 0.001, *d* = 8.23). The results suggested that the degree of self-relevance was operationalized successfully.

**TABLE 1:** Mean IOS scores, helping proportion, decision time, and affective rating scores under different conditions in Study 1.

	**Friend**	**Acquaintance**	**Stranger**
IOS	5.25 ± 0.48	3.35 ± 0.50	1.38 ± 0.46
Helping rates (%)	81.24 ± 16.85	68.15 ± 19.84	55.58 ± 20.96
Decision times (ms)	297.43 ± 110.59	319.48 ± 129.62	356.23 ± 134.90
Unpleasant scores	3.85 ± 0.33	3.01 ± 0.42	2.15 ± 0.36

#### Helping Rates

**FIGURE 2:**
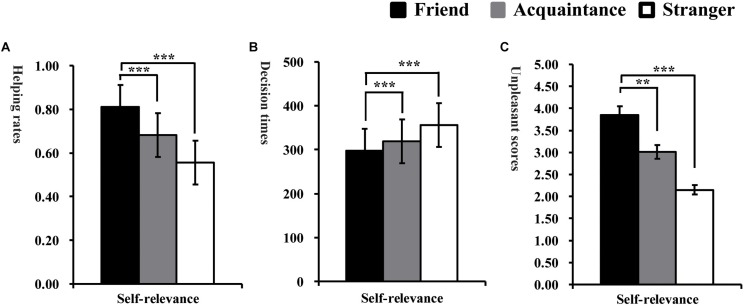
Average helping rates **(A)**, decision times **(B)**, and unpleasantness scores **(C)** when making decisions toward friends, acquaintances and strangers in Study 1. ^∗^*P* < 0.05, ^∗∗^*P* < 0.01, and ^∗∗∗^*P* < 0.001.

The overall percentages of trials in which participants selected help were 73.44% (*SD* = 22.22%). A one-sample *t*-test indicated that the average help rates were significantly higher than the manipulated 50% reinforcement rates, *t*(71) = 10.97, *p* < 0.001, suggesting that participants trended toward deciding to help the protagonists in the dilemmas. To test our hypothesis that self-relevance affects the behavioral responses of moral decision in the helping dilemmas, the proportion of help choices and response times were subjected to repeated-measures ANOVA according to the experimental design (see Figure 2). The analysis revealed a main effect of self-relevance, [*F*(2, 142) = 66.88, *p* < 0.001, $\eta_{p}^{2}$ = 0.49]. Multiple comparisons by the Bonferroni corrections showed that participants made more helping decisions toward friends (*M* = 81.24%, *SD* = 16.85%) than toward acquaintances (*M* = 68.15%, *SD* = 19.84%, *p* < 0.001, *d* = 0.71) and strangers (*M* = 55.58%, *SD* = 20.96%, *p* < 0.001, *d* = 1.35) and made more helping decisions toward acquaintances than toward strangers (*p* < 0.001, *d* = 0.62).

#### Decision Times

ANOVA for the mean response time indicated that participants made decisions faster toward friends (*M* = 297.43 ms, *SD* = 110.59 ms) than acquaintances (*M* = 319.48 ms, *SD* = 129.62 ms, *p* < 0.001, *d* = −0.18) and strangers (*M* = 356.23 ms, *SD* = 134.90, *p* < 0.001, *d* = −0.48) and made decisions faster toward acquaintances than strangers (*p* < 0.001, *d* = −0.28), *F*(2, 142) = 27.82, *p* < 0.001, $\eta_{p}^{2}$ = 0.28.

#### Unpleasant Scores

ANOVA conducted on the subjective unpleasure ratings scores indicated that participants reported significantly more unpleasantness for the decisions for strangers (*M* = 2.15, *SD* = 0.36) than friends (*M* = 3.85, *SD* = 0.33, *p* < 0.001, *d* = −4.92) and acquaintances (*M* = 3.14, *SD* = 0.68, *p* < 0.01, *d* = −1.82), as well as significantly more unpleasant for acquaintances than a friend (*p* < 0.05, *d* = −1.32), *F*(2, 142) = 24.87, *p* < 0.01, $\eta_{p}^{2}$ = 0.51. All types of decisions were rated as highly unpleasant.

Study 1 showed that self-relevance indeed affects behavioral responses during moral decision making, such as altruistic helping choices, decision times and subjective negative emotional responses. Compared to strangers, participants made more helping choices along with reduced response times and subsequently experienced less unpleasantness for friends and acquaintances. In accordance with previous studies, people could make more altruistic behaviors for close others (e.g., relatives, friends, and acquaintances) compared strangers, such as helping (O’Neill and Petrinovich, 1998), empathy (Reniers et al., 2012; Juan et al., 2016) and cooperation (Chen et al., 2017). Some studies also suggested that moral decision making toward intimate others seems to elicit less dilemma conflict and was made more quickly compared to strangers (Bloomfield, 2008; Chen et al., 2009; Crockett et al., 2014). In this study, friend and acquaintance are more intimate with subjects compared to stranger and thus deserved faster and more help. We have speculated that this differential altruistic moral decision may be caused by the participants’ experience of different levels of dilemma conflict and aversion when making moral decisions toward others with different self-relevance. As expected, participants have reported stronger subjective unpleasant experience when making moral decisions toward friends and acquaintances compared to strangers. Previous studies have also suggested that moral decision making usually evoked strong moral conflict and aversion due to series of dilemma trade-offs between self and others about gains and losses (Greene and Haidt, 2002; Crockett, 2016; Pletti et al., 2016). We speculated that individuals may make altruistic moral choices in order to alleviate such conflicts and aversions. In fact, we have indeed found that participants subjectively reported strong unpleasant experiences while making moral decision about whether to help others by sacrificing self-interest. Therefore, compared with friends, people may experience stronger moral conflict and aversions when deciding whether to help strangers due to taking more attentional resources and cognitive effort to weigh gains and losses between self and strangers. In conclusion, these results suggested that the closer the decision makers’ perceived self-relevance with the targeted other was, the weaker the dilemma conflicts and negative emotional responses experienced were, and the more effectively the dilemma conflicts were solved.

More important, previous studies have reported that moral decision making was also affected by external risk information, similar with economic decision making (Tobler et al., 2008; Shenhav and Greene, 2010). For example, recent studies revealed that the interpersonal reputational loss risk can promote participants to make more altruistic decisions (Bixter and Luhmann, 2014; Arfer et al., 2015). Moreover, previous studies also indicated that self-relevance can influence risk decision-making (Zhao et al., 2013; Raue et al., 2015; Zhang et al., 2018). These studies suggested that moral decision making may be influenced by the interaction between self-relevance and reputational loss risk. Thus, the next study would reveal the processing of trade-offs between self- and other-interest under the interacted influence of both self-relevance and reputational loss risk.

## Study 2

In Study 1, we found that participants made more helping choices for friends and acquaintances compared to strangers, along with shorter decision times and weaker subjective unpleasant experience under the costly help dilemmas. Study 2 used a more complex task with interpersonal interaction to examine how reputational loss risk effects interact with social-distance effects. In Study 2, there was a 3 (self-relevance: friend, acquaintance, and stranger) × 2 (reputational loss risk: high and low risk) within-subject experiment. We expected participants to make more altruistic choices for friends compared with acquaintances and strangers, but this differential altruistic moral decision was weakened under the high risk of reputational loss.

### Methods

#### Participants

According to the experimental design of study 2, a power analysis (G^∗^Power 3.1) suggested that 50 participants would ensure 90% statistical power even in the case of small-to-medium effect size. Therefore, we recruited 71 participants (36 males, 35 females), ranging in age from 18 to 25 (*M* = 23.65). The experiment was also conducted in accordance with the Declaration of Helsinki and was also approved by the Ethics Committee of Hunan Normal University.

### Materials and Procedure

The moral decision-making dilemma was also used in Study 2. Unlike Study 1, Study 2 consisted of high and low reputational loss risk conditions. Under the high reputational loss risk conditions, participants were told their choices would be sent over the Internet to another computer in the next room for target persons (i.e., friends, acquaintances or stranger) to see in 95% of trials. However, under the low reputational loss risk conditions, participants were told that only in 5% of trilas would their choices be sent to target persons to see. Previous studies suggested that participants would experience reputational concern when their choices are likely to be known to the target persons (Bixter and Luhmann, 2014; Arfer et al., 2015). In summary, Study 2 had six types of moral decision making: high risk level-friend, low risk level-friend, high risk level-acquaintance, low risk level-acquaintance, high risk level-stranger, low risk level-stranger, for a total of 180 trials.

#### Procedure

Firstly, participants completed the IOS, which was described as an interpersonal relationship measure. Then the experimenter explained the moral decision making under high and low risk of reputational loss conditions orally. Participants were told the task had three bystanders in the next room and they have a kind of probability to see their own choices on the other computer. In detail, the probability was presented in words between each dilemma and its options, such as “The probability of being known is 95%” or “The probability of being known is 5%.” After participants completed moral decision-making, they were paid 10 Yuan RMB ($1.45).

### Results and Discussion

#### IOS Scores

We analyzed the results of the scores of IOS for target persons presented by each participant. ANOVA indicated that there was a significant main effect of self-relevance, *F*(2, 140) = 258.22, *p* < 0.001, $\eta_{p}^{2}$ = 0.74, suggesting that the IOS scores between self and friend (*M* = 5.26, *SD* = 0.21) were higher than those between self and acquaintance (*M* = 3.10, *SD* = 0.19, *p* < 0.001, *d* = 10.79) and those between self and acquaintance were higher than those between self and stranger (*M* = 1.25, *SD* = 0.22, *p* < 0.001, *d* = 18.65). The results suggested that the degree of self-relevance was operationalized successfully in Study 2 (see Table 2).

**TABLE 2:** Mean IOS scores, helping proportion, decision time, and affective rating scores under different conditions in Study 2.

		**Helping rates**	**Decision times**	**Unpleasant scores**
High	Friend	85.55 ± 12.23	384.91 ± 175.67	3.55 ± 0.24
reputational	Acquaintance	74.21 ± 16.16	396.33 ± 200.30	1.86 ± 0.28
loss risk	Stranger	59.42 ± 20.11	405.10 ± 218.54	2.94 ± 0.24
Low	Friend	79.14 ± 14.60	348.55 ± 146.68	3.93 ± 0.21
reputational	Acquaintance	62.77 ± 18.58	411.93 ± 185.72	2.20 ± 0.25
loss risk	Stranger	45.10 ± 20.04	370.92 ± 178.75	3.46 ± 0.27

#### Helping Rates

**FIGURE 3:**
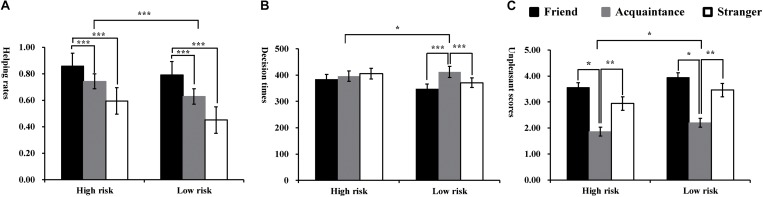
Average helping rates **(A)**, decision times **(B)**, and unpleasantness scores **(C)** when making decisions toward friends, acquaintances and strangers under high and low reputational loss risks conditions in Study 2. ^∗^*P* < 0.05, ^∗∗^*P* < 0.01, and ^∗∗∗^*P* < 0.001.

The descriptive results (Average and standard deviations) of proportions of help choices, decision times, and the subjective unpleasant rating are presented in Table 2. The overall percentage of trials in which participants selected help was 68% (*SD* = 19.28%). A one-sample *t*-test indicated that the average help rates were significantly higher than the manipulated 50% reinforcement rates, *t*(70) = 6.26, *p* < 0.001, suggesting that participants trended toward deciding to help the protagonists in the dilemmas. The helping proportions and response times were subjected to repeated-measures ANOVA according to the experimental design (see Figure 3).

Results indicated that there were main effects of self-relevance [*F*(2, 140) = 103.59, *p* < 0.001, $\eta_{p}^{2}$ = 0.60] and risk levels [*F*(1, 140) = 73.98, *p* < 0.001, $\eta_{p}^{2}$ = 0.51] on helping proportion. There was a descending effect for helping proportion showing that participants made more helping decisions toward friends (*M* = 82.30%, *SD* = 13.21%) than toward acquaintances (*M* = 68.50%, *SD* = 17.42%, *p* < 0.001, *d* = 0.89) and strangers (*M* = 52.30%, *SD* = 20.16%, *p* < 0.001, *d* = 1.76) and made more helping decisions toward acquaintances than toward strangers (*p* < 0.001, *d* = 0.86). Participants made more helping decisions under high risk levels (*M* = 73.10%, *SD* = 15.46%) compared to low risk levels (*M* = 62.30%, *SD* = 16.52%, *d* = −68). Moreover, the interaction effect between self-relevance and risk levels at the helping rates was significant [*F*(2, 140) = 7.60, *p* < 0.01, $\eta_{p}^{2}$ = 0.16]. Simple effect analysis revealed that there was a significant difference of helping choices toward three target persons under low risk levels [*F*(2, 140) = 97.88, *p* < 0.01], showing a helping proportion descending effect reflected as “friend > acquaintance > stranger” (*ps* > 0.01). Furthermore, such a descending effect was weakened under high risk levels [*F*(2, 140) = 69.42, *p* < 0.01]. Additionally, participants made more helping choices toward friends under high than low risk level conditions [*F*(1, 70) = 21.85, *p* < 0.001]. However, such differences of helping proportions between high and low risk levels were larger toward both acquaintances [*F*(1, 70) = 37.16, *p* < 0.001] and strangers [*F*(1, 70) = 58.82, *p* < 0.001].

#### Decision Times

Meanwhile, there were main effects of self-relevance [*F*(2, 140) = 11.43, *p* < 0.001, $\eta_{p}^{2}$ = 0.14] and risk levels [*F*(1, 140) = 5.89, *p* < 0.05, $\eta_{p}^{2}$ = 0.11] on the response times. Participants made slower decisions toward acquaintances (*M* = 404.12 ms, *SD* = 122.02) than toward both friends (*M* = 366.73 ms, *SD* = 108.68, *p* < 0.001, *d* = 0.32) and strangers (*M* = 388.01 ms, *SD* = 123.12, *p* < 0.05, *d* = 0.13) as well as slower decisions toward strangers than toward friends (*p* < 0.05, *d* = 0.18). Participants made slower decisions under high risk levels (*M* = 395.45 ms, *SD* = 204.45 ms) compared to low risk levels (*M* = 377.13, *SD* = 181.36 ms, *p* < 0.05, *d* = 0.10). Moreover, the interaction effect between self-relevance and risk levels was significant at the response times, *F*(2, 140) = 7.86, *p* < 0.01, $\eta_{p}^{2}$ = 0.10. Simple effect analysis revealed that there was no significant difference toward the three targets (friend: *M* = 384.91 ms, *SD* = 175.67 ms; acquaintance: *M* = 396.33 ms, *SD* = 200.30 ms; stranger: *M* = 405.10 ms, *SD* = 218.54 ms) under high risk levels [*F*(2, 140) = 1.99, *p* > 0.14]. However, there was a significant difference toward the three target persons under low risk levels [*F*(2, 140) = 18.97, *p* < 0.001], showing that participants made slower decisions toward acquaintances (*M* = 411.91 ms, *SD* = 185.72 ms) than toward both friends (*M* = 348.55 ms, *SD* = 146.68 ms, *p* < 0.01, *d* = 0.38) and strangers (*M* = 370.92 ms, *SD* = 178.75 ms, *p* < 0.01, *d* = 0.22) as well as slower decisions toward strangers than toward friends (*p* < 0.05, *d* = 0.14). Additionally, participants made slower decisions under high than low risk levels toward friends [*F*(1, 70) = 16.65, *p* < 0.001] and strangers [*F*(1, 70) = 10.92 *p* < 0.01]. However, such differences in decision times between high and low risk levels was not observed for acquaintances [*F*(1, 70) = 1.50, *p* > 0.05].

#### Unpleasantness Rating Scores

ANOVA indicated that participants reported significantly stronger unpleasant experiences for the decisions for acquaintances (*M* = 2.03, *SD* = 0.22) than friends (*M* = 3.74, *SD* = 0.26, *p* < 0.05, *d* = −7.10) and strangers (*M* = 3.20, *SD* = 0.24, *p* < 0.01, *d* = −5.08) but no significant difference between friends and strangers (*p* > 0.05), *F*(2, 140) = 5.78, *p* < 0.05, $\eta_{p}^{2}$ = 0.23. Moreover, participants reported significantly stronger unpleasant experiences for the decisions under high than low risk levels, *F*(1, 70) = 4.88, *p* < 0.05, $\eta_{p}^{2}$ = 0.16. However, the interactions between self-relevance and risk levels was not significant, *F*(2, 140) = 1.26, *p* > 0.05. All types of decisions were rated as highly unpleasant experiences.

In accordance with study 1, participants also showed an obvious differential altruistic tendency at helping proportions in Study 2, which reflected as “friend > acquaintance > stranger.” Moreover, an interesting “acquaintance effect” was observed at the decision times and subjective unpleasant rating scores, which indicated that participants took more time to make helping choices toward acquaintances and subsequently reported a stronger averse experience compared to friends and strangers. This finding maybe arose from the fact that predicting how our decisions will affect others is inherently uncertain (Harsanyi, 1977 and Research). Some Chinese psychological studies suggested that acquaintances elicited the largest affective and cognitive uncertainties compared with family members, friends, and strangers during interpersonal interaction (Huang, 2010; Yuan and Guo, 2017). In addition, the results of Study 2 also showed that compared with under the low risk of reputational loss, participants made more and slower helping choices under the high risk of reputational loss. Moreover, this promotion was larger toward acquaintances and strangers compared with friends. This finding was consistent with previous studies which reported that subjects were more prosocial during moral-hazard gambling tasks when their choices would be seen by their partners (Bixter and Luhmann, 2014; Arfer et al., 2015). Therefore, these findings suggested that reputational concerns were a key restraint on egoistic exploitation under moral hazard. In this study, participants indeed made larger improvements regarding their choice to help more distant others (acquaintance and stranger) to maintain a good moral self-image.

## General Discussion

### Egoistically Biased Altruistic Moral Decision Making

Results from Studies 1 and 2 found that all participants preferred to be willing to sacrifice self-interest to help others under moral dilemmas. This finding was consistent with the findings of previous studies which reported that people are generally altruistic, such as trusting and helping others and cooperating with others (Loke et al., 2011; Wang et al., 2016). However, this general altruistic tendency was based on egoism, which indicated that helping choices represented a decreasing pattern as “friend > acquaintance > stranger” in our study. This result was supported by numerous previous behavioral studies. For example, Olson and Cognition (2008) revealed that children’s compliance with fairness and justice were influenced by the object of behavior (familiar persons and strangers), reflecting that they were willing to allocate more things for their family members and friends. Liviatan et al. (2008) showed that participants judged that it was not allowed and unfair for strangers to cheat in the exam, but they judged that it was allowed and important for their friends to cheat in the exam. A recent seminal psychological study has demonstrated participants’ willingness to forego reward to spare others from harm as well as strongly egoistic tendencies in participants’ unwillingness to harm themselves for others’ benefit (Volz et al., 2017). In addition, Rai and Fiske (2011) have posed the moral relationship regulation theory to uncover the influence of interpersonal relationship on moral behaviors. Specifically, this theory argued that moral principle guiding intersubject moral behaviors between self and others depended on the structure of the interpersonal relationship in a specific moral situation, which was reflected as psychological distance between decision makers and others in moral dilemmas. Therefore, results suggested that people made egoistically biased altruistic moral decision-making in the costly helping dilemmas.

### Uncertainty in Moral Decision Making Toward Acquaintance Evokes More Reflection and Stronger Aversion

More interestingly, Study 2 observed an evident “acquaintance effect” at decision times and subjective affective rating under the low risk of reputational loss. In detail, participants took more time to think and make decisions toward acquaintances and subsequently experienced stronger unpleasant. This finding was supported by many previous studies. For instance, a Chinese behavioral study reported that in the explicit and implicit judgment of interpersonal affective preference, individuals hold a positive affective preference to themselves, a negative affective preference to strangers, and a fuzzy affective preference to acquaintances (Yuan and Guo, 2017). According to the Chinese regularities of interpersonal relationships from Huang (2010), acquaintances would develop into either friends or strangers over time, and thus there are many uncertainties. This finding was supported by previous studies which suggested that uncertainty in decision making is generally reflected in longer response times (Pleskac and Busemeyer, 2010; De Martino et al., 2013). This explanation indicated that participants took longer times to weigh gains and losses between self and others. Moreover, participants reported stronger unpleasantness after each dilemma decision toward acquaintances. An abundance of relevant studies reported that moral decision making can evoke strong aversive experience (Greene, 2009; Sarlo et al., 2012, 2014; Pletti et al., 2015). Thus, the “acquaintance effect” in decision times and subjective affective rating suggested that moral decision making toward acquaintances elicited stronger moral conflict, and participants allocated more cognitive efforts to resolve it. Otherwise, this effect was not observed in western participants according to the previous studies. In detail, the western self is usually described as an independent self-construal, which suggests that most of their interpersonal relationships were classified according to self and non-self, while they were not sensitive to the classification of acquaintances according to the Chinese relational self (Cross et al., 2000; Markus and Kitayama, 2010; Uskul and Over, 2018). For example, Longhi et al. (2011) have explored the cultural differences between independent and interdependent self-construal during self-face recognition. Results suggested that Chinese participants showed a “boss effect” which indicating that Chinese participants can faster recognize their boss’ face compared to their own face. However, European American participants did not show a “boss effect” and maintained the self-face advantage even in the presence of their supervisor’s face.

### Reputational Concerns Motivate People to Restrain Self-Interest

Study 2 also showed that participants took more time to make more helping choices under the high risk of reputational loss condition. Importantly, reputational concerns can motivate participants to restrain the egoistically altruistic tendency, which stemmed from participants’ larger improvement of helping choices toward acquaintances and strangers compared to friends. Moreover, decision times among the three target persons were not significantly different. It has been proposed that improving ones’ reputation was a particularly important way that prosocial behavior can ultimately serve self-interest (Fehr and Fischbacher, 2003). Alexander (1986) has proposed indirect reciprocity to explain the origin of human morality and large-scale cooperative behavior. A subsequent larger number of studies have demonstrated that the reputational mechanism was one important mechanism of human indirect reciprocity, such as image score (Nowak and Sigmund, 1998), standing (Hamlin and Sugden, 2004; Brandt and Sigmund, 2006), and tag (Riolo et al., 2001). For example, Sylwester and Roberts (2010) found that participants who were more generous in the first round were chosen by partners who were more generous in the second round of an economic game. They speculated that these more generous subjects had invested in their reputation. Other studies have reported that an audience-dependent generosity have been particularly observed among participants who were asked in the presence of others to volunteer for charities (Bereczkei et al., 2007, 2015; Simpson and Willer, 2008). In Study 2, participants who were told that their choice results would be presented to target persons indeed made more helping choices and took longer times to weigh gains and losses between self and others. Thus, results suggested that reputational concerns played a key role in promoting altruistic moral decision-making.

Additionally, there are at least two limitations to this study. The first aspect resides in the fact that the moral dilemmas were assumed and imagined, and college students lacked direct experiences although they could understand these scenarios. Thus, we suggest that research about moral decision making should be conducted under some real and daily moral dilemmas. The second limitation is that our operation approach for reputational loss risk may not exclude the influences of social desirability on participants’ decisions. Participants may still experience reputational pressure from the experimenter in addition to protagonist under dilemmas due to the lack of anonymity during moral decision making.

## Conclusion

In summary, our findings provide important evidence that both self-relevance and reputational loss risk have a robust interacted effect on moral decision making in the costly helping dilemmas. Our studies provide a research perspective to reveal the cognitive mechanism of moral decision making under the interaction between internal and external factors. The results not only extend moral relationship regulation theory (Rai and Fiske, 2011) and reputational mechanism (Alexander, 1986) but describe the egoistically biased altruistic moral decision making from the emotional and cognitive perspectives according to the dual-process theory. In conclusion, these findings indicate that people have an egoistically biased altruistic tendency, and reputational concerns rather than altruism motivate restrained self-interest during moral decision making in costly helping dilemmas.

## Data Availability Statement

The datasets generated for this study are available on request to the corresponding author.

## Ethics Statement

The studies involving human participants were reviewed and approved by the Hunan Normal University. The patients/participants provided their written informed consent to participate in this study.

## Author Contributions

YLZ and XX prepared the materials and measures. JL and HZ collected and analyzed the data. QT and SZ drawn Tables and Figures. YLZ and YO wrote and amended the manuscript. YPZ reviewed the manuscript.

## Conflict of Interest

The authors declare that the research was conducted in the absence of any commercial or financial relationships that could be construed as a potential conflict of interest.
